# Trends in the Application of “Omics” to Ecotoxicology and Stress Ecology

**DOI:** 10.3390/genes12101481

**Published:** 2021-09-23

**Authors:** Joshua Niklas Ebner

**Affiliations:** Spring Ecology Research Group, Department of Environmental Sciences, University of Basel, 4056 Basel, Switzerland; joshua.ebner@unibas.ch

**Keywords:** ecotoxicology, stress ecology, omics, trends, technology, taxa, cross-species, review

## Abstract

Our ability to predict and assess how environmental changes such as pollution and climate change affect components of the Earth’s biome is of paramount importance. This need positioned the fields of ecotoxicology and stress ecology at the center of environmental monitoring efforts. Advances in these interdisciplinary fields depend not only on conceptual leaps but also on technological advances and data integration. High-throughput “omics” technologies enabled the measurement of molecular changes at virtually all levels of an organism’s biological organization and thus continue to influence how the impacts of stressors are understood. This bibliometric review describes literature trends (2000–2020) that indicate that more different stressors than species are studied each year but that only a few stressors have been studied in more than two phyla. At the same time, the molecular responses of a diverse set of non-model species have been investigated, but cross-species comparisons are still rare. While transcriptomics studies dominated until 2016, a shift towards proteomics and multiomics studies is apparent. There is now a wealth of data at functional omics levels from many phylogenetically diverse species. This review, therefore, addresses the question of how to integrate omics information across species.

## 1. Introduction

The main objective of ecotoxicology and stress ecology is to understand and predict the effects of contaminants and environmental stressors on ecological systems [1,2]. These research fields are interrelated since toxicants often interact with “natural” stress factors such as temperature and nutritional status [3]. Exposure of organisms to such stressors triggers a series of cascading changes at multiple levels of the molecular hierarchy [4]. Since molecular changes inform about potential negative impacts following exposure, their detection and measurement can reveal if organisms are exposed to pollutants and, when determined experimentally, how they might respond following exposure in their natural habitat. Regulatory molecular pathways involved in these responses exhibit changes of levels, interactions, and feedback loops of (bio)molecules of different types active in networks with increasing complexity [5,6]. High-throughput methods allow the simultaneous quantification and characterization of network components (e.g., transcripts, proteins, and metabolites). Collectively, these methods are referred to as “omics”, with the aim to address biological processes as integrated and interacting systems. Components of these systems comprise very different physicochemical properties and exhibit complex nonlinear interactions [7]. Despite this complexity, improvements in technologies for measuring molecular-level endpoints now provide high-resolution information on molecular networks and an impetus for re-evaluating the ability to incorporate these measurements into modern-day risk assessment procedures [8,9,10,11,12]. Driven by the developments in genomics and a systems-oriented perspective on biology, the quest for unbiased identification of biomarkers and relevant pathways has, arguably, already been transformed [9]. Omics applications generate a wealth of data that researchers can integrate with adverse outcome pathways (AOPs) to establish links between sub-individual biomarker responses and potential effects at higher levels of biological organization [13,14]. For example, core networks of transcripts and signaling pathways that respond to estrogen exposure have been identified across six independent laboratories [15]. Omics assessments do not necessarily require prior assumptions about the choice of biomarkers and provide an unbiased picture of the ecotoxicological effect at an early stage [8,9]. Consequently, ecotoxicological tests are being supplemented by making the best use of these methods and addressing biological traits at different degrees of complexity [15,16,17,18].

Because it is only possible to test the effects of chemicals and other stressors on a restricted range of species and exposure scenarios, researchers are faced with a significant challenge of how to translate the measurements in model species exposed to model stressors into predictions of impacts for a broader range of species and ecosystems. It is impractical to determine all molecular effects for each stressor found in the environment (and their synergistic and antagonistic effects) for each species found on planet Earth (~8.7 million eukaryotic species globally) [4,19]. Since taxa and individual species within classes and families often harbor similar genomic architectures and conserved cellular pathways, knowledge about available data is greatly beneficial for extrapolating results [20,21,22,23]. Furthermore, the knowledge of identified responses following stressor exposure in one organismal group may be used to predict the modes of action of similar agents in other groups [24]. Thus, so-called bridging effects may be identified that can be used in extrapolation to other taxa, greatly accelerating our capability to evaluate the environmental impact on a diverse set of species over time [25]. For practitioners and researchers planning to apply these methods, or wanting to identify conserved cross-species responses, understanding which methods are most frequently employed, which taxa, species and stressors have been investigated most frequently and which levels of biological organization multiomics studies typically investigate may help broaden the application of omics technologies to novel taxa and aid choice of study system and omics layer. This knowledge may also help avoid the recurring investigation of the same species and stressors. Accordingly, this review identifies such trends, which may guide the choice of model taxa and stressors(s) that have not yet been studied or for which no systems biological data on specific omics levels have been generated.

## 2. Materials and Methods

Peer-reviewed studies (2000–2020) were identified based on an extensive literature search using Google Scholar and Web of Science with a combination (using the Boolean operator “AND”) of the following keywords: “Omic *” “Ecotox *”, with the keyword “Omic *” iteratively replaced by “Proteom *”, “Transcriptom *”, “Metabolom *”, or “Multi Omic *”. Asterisk (*) represent any character, group of characters, or no character to increase the search space. Additionally, reference lists of recent (2018–2020) literature reviews were scanned, and the Daphnia Stressor Database searched for ecotoxicological and stress ecological studies [26]. Studies on (epi)genomics, lipidomics, plant systems, and cell cultures were omitted from the analyses. Developments in these fields have been reviewed elsewhere [27,28,29,30,31,32,33]. Genomics studies in Evolutionary Toxicology that e.g., determine changes in allelic or genotypic frequencies caused by increased mutation rates are also covered elsewhere [34,35,36,37,38,39]. Included studies that utilized one or more of the following omics layers: transcriptomics, proteomics, metabolomics, and multiomics to study the effects of one or multiple stressors were classified based on (i) omics layer, (ii) studied species/taxa, and (iii) studied stressor(s). Data are available in Table S1 and were summarized and visualized in R v.4.1.0 (R Core Development Team, 2021).

## 3. Results

**Figure 1:**
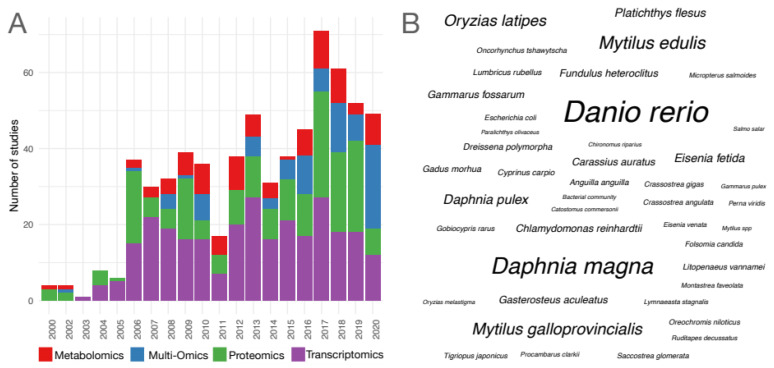
(**A**) Ecotoxicological and stress ecological studies between 2000 and 2020 that used one or multiple omics methods to investigate molecular changes following exposure to (environmental and chemical) stressors. (**B**) Word cloud showing representative model and non-model species studied across all years (only species with n > 2 studies are shown). Word size corresponds to the number of studies.

A total of 648 studies were included, representing many stressors (n = 259) and species (n = 184). Transcriptomics was the most frequently applied method (43%), followed by proteomics (30%), metabolomics (13%) and finally, multiomics (13%). While the number of transcriptomic studies stayed constant across the years, there is a trend towards increased usage of proteomics, with studies dominating the literature from 2017 to 2019 (40% proteomics vs. 34% transcriptomics). Furthermore, proteomics is not less frequently used in multiomics studies as stated previously [40]. It was the second most frequently applied method overall and out of 84 included multiomics studies, 62% used proteomics in concert with at least one other omics layer. A trend towards combining omics technologies to investigate the impact of stressors on organisms was observed, with multiomics studies making up the majority (44%) of the literature in 2020 (Figure 1A). Across all years, multiomics studies most frequently used a combination of transcriptomics and proteomics (38%), followed by transcriptomics and metabolomics (33%) and proteomics and metabolomics (21%). Yet, multiomics data sets beyond two layers are still rare.

**Figure 2:**
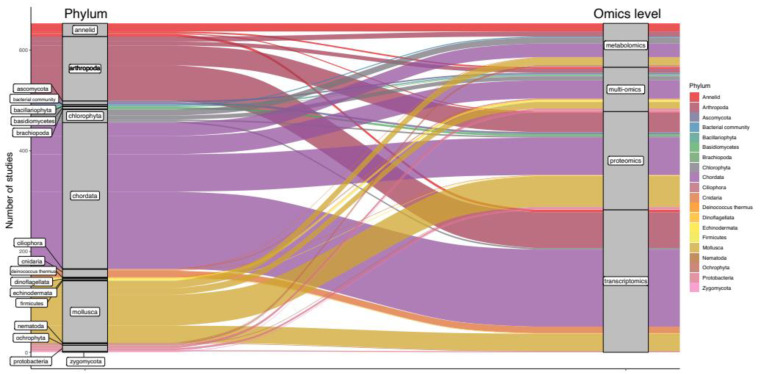
Alluvial diagram depicting the relative frequencies of phyla (left blocks) for which data on different omics levels (right blocks) have been generated between 2000 and 2020. Stream fields between the blocks are color coded based on taxonomic affiliation and represent the total number of times specific omics methods have been applied within the specified phyla.

Out of 184 investigated species, the five most frequently studied organisms were *Danio rerio* (11%), *Daphnia magna* (7%), *Mytilus edulis* (4%), *Oryzias latipes* (3%), *Pimephales promelas* (3%) and *Oncorhynchus mykiss* (3%) (Figure 1B). Notably, most studies focused on chordata (44%, Figure 2), potentially since approximately 70% of human protein-coding genes, including disease-associated genes, have an ortholog in fish [41]. Except for a few phyla, there is little preference for using specific omics methods for any one phylum, indicating a balanced investigation at different levels of the molecular hierarchy (Figure 2).The relative number of proteomics studies in mollusca was higher than for any other phylum, indicating a preference for investigating protein-level responses in *Mytilus* (Figure 2). Arthropods were the second most frequently studied phylum (19%, Figure 2), with *Daphnia magna* and *D. pulex* being the species of choice when investigating the adverse effects of environmental stressors in this phylum [42,43]. Studies probing the toxicobiology of the amphipod *Gammarus fossarum* have also become more common. For these species, high-quality sequence information such as sex-specific transcriptomes and genomes are publicly available, facilitating the analysis and integration of data at various omics levels [44,45,46,47]. Within the chlorophyta, *Chlamydomonas reinhardtii* was by far the most often investigated organism, potentially owing to established laboratory cultivation protocols and available sequencing information [48,49]. Within the cnidaria, *Orbicella* (previously *Montastrea) faveolata* was the most often investigated organism. A wide range of sequencing data is available for this endangered reef-building coral, from whole-genome assemblies to RNA-seq data [50,51,52]. Notably, there is little information on proteomic, metabolomic, and multiomic responses within the cnidaria, indicating a knowledge gap for molecular responses in cnidaria beyond the transcriptional level (Figure 2). Within the mollusca, *Mytilus edulis* and *M. galloprovincialis* were the most frequently investigated species (21% and 16%, respectively). Representing 5% of studies within this phylum, the invasive mollusk *Dreissena polymorpha* represents a counterpart to Mytilus to monitor inland freshwater bodies. Within the fungi, *Aspergillus niger* (ascomycota), and *Pleurotus ostreatus* (basidiomycetes) may become representative sentinel species since their molecular biology is actively investigated [53,54].

**Figure 3:**
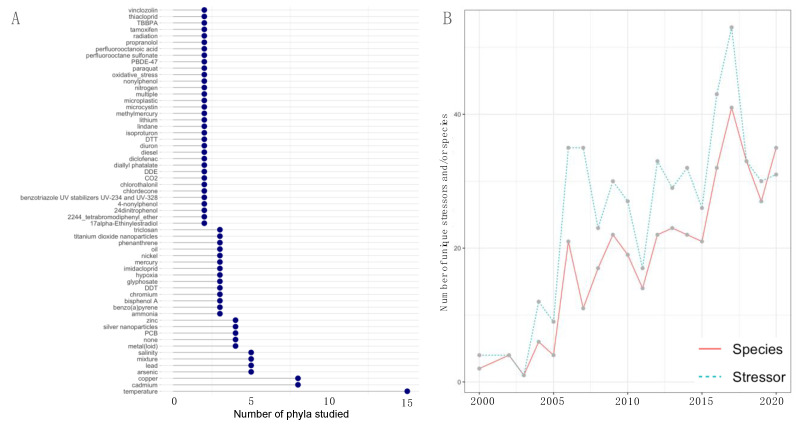
Overview of stressors, phyla and species investigated using omics methods from 2000 to 2020. (**A**) Number of phyla studied for unique stressors (only stressors for which more than one phylum has been investigated are shown). (**B**) Line plot showing the number of unique stressors and species studied each year.

For the first time in 2020, more different species than stressors were studied, indicating that omics technologies are now being applied to a broader variety of species than stressors (Figure 3B). The number of unique stressors investigated each year was higher than the number of unique species used as study systems (Figure 3B). The most frequently studied stressors were temperature (8%), 17α-Ethinyl estradiol (8%), cadmium (5%), copper (2%), oil (2%), bisphenol A (0.15%) and silver nanoparticles (0.13%). Interestingly, most studies (11%) tested the adverse effects of chemical mixtures in situ, most often in the form of wastewater effluents (WWE) and via in situ studies that compared polluted with nonpolluted reference sites. These studies most often employed transcriptomics (48%) followed by proteomics (29%) and metabolomics (18%).

Except for 17α-Ethinyl estradiol, the most frequently studied stressors were also studied in the most phyla (Figure 3A). For example, the effects of temperature were studied in 15 different phyla compared to any other stressor and most frequently in arthropods (21%), followed by cnidaria (19%) and mollusca (12%). In contrast, the adverse effects of 17α-Ethinyl estradiol have almost exclusively been studied in chordata (96%), indicating a knowledge gap in the impacts of 17α-Ethinyl estradiol on other taxonomic groups. Additionally, the effects of 17α-Ethinyl estradiol have almost exclusively been studied on the transcriptomic level (Figure S1). In contrast, the effects of temperature have been studied using a broad range of methods, most notably via many multiomics studies (Figure S1). The adverse effects of heavy metals such as cadmium and copper have been studied most often in arthropods (38% and 22% respectively), mollusca (27% and 27%), and chordata (16% and 22%). The impacts of these heavy metals have also been studied using a broad range of methods, except for zinc, for which primarily transcriptomic studies have been conducted. An overview of which omics methods have been applied to which stressor is given in Figure S1.

## 4. Discussion

It is beyond the scope of this article to highlight all trends and challenges associated with the application of omics in ecotoxicology and stress ecology. For example, integrating information across multiple omics layers requires addressing a multitude of challenges [55,56]. Additional issues arise from the complexity of life and environmental variability. A species may not respond uniformly across its range, since populations may react differentially to environmental change [57,58,59]. The increased application of proteomics and multiomics in the literature suggests the importance of studying functional changes gained from these analyses since they may offer a more informative perspective of toxicopathic effects compared to e.g., mRNA copy numbers [60,61,62,63,64]. Although arguments have been made to prioritize working on model organisms with complete genome data available [11], the molecular responses following stressor exposure are increasingly being studied in phylogenetically and ecologically diverse non-model species. An important consideration is how measurements in these species can be translated into predictions of impacts for a wider range of species and ecosystems. Taken together, the growing availability of functional omics data on a wide variety of non-model species calls for cross-species comparisons based on standardized functional annotation. Identifying evolutionarily conserved expression patterns may be a promising path forward. Knowledge of these conserved pathways enables the detection of a pollutant in any ecosystem and the determination of the effects of environmental change on novel species based on previously obtained data [65,66,67]. For example, exposure to hexahydro-1,3,5-trinitro-1,3,5-triazine (RDX) elicited a conserved mode of action throughout phylogenetically remote organisms [65]. Identifying conserved expression patterns (or pathways of toxicity (PoT) [12]) requires concerted functional analyses based on gene set enrichment, network modeling, text mining, graph-based, and pathway enrichment analyses [68,69,70,71,72,73,74,75]. For example, in the field of phenomics, ontology-based semantic mapping (OS-Mapping) has been used to identify chemical by species interactions and similar mechanisms of action (MOAs) across species [76,77]. Here, phenotypes are described as free text and are then made computable by annotating them with appropriate ontology terms (e.g., Gene Ontology (GO), Pfam, FunCat, KEGG, and WikiPathways [78,79,80,81,82,83]). A significant advantage of this approach is that functional datasets are less concerned with comparing absolute changes of (bio)molecules between studies but are focused on how the function of the biological system changes. Ultimately, a systems approach with a functional perspective gains relevance and meaning [84,85,86,87,88]. Considering that a prevalent argument against the adoption of omics in chemical risk assessments is the lack of simplicity of use [89], presenting results from a functional approach in “human-readable language” may facilitate the incorporation of omics in environmental monitoring efforts and link pathway perturbations to the phenotype [90].

This article highlights the available wealth of disparate information on systems molecular responses in a large variety of species and stressors and with sufficient mode of action data that allows scanning for patterns from which general patterns, rules, theory, and models can be inferred. An important consideration is the storage and dissemination of findings based upon a commonly agreed ontology and structured vocabulary to facilitate data-sharing and mining. For example, the diXA project has assembled a set of toxicogenomics studies from diverse sources, which in part comprise multiomics data [91]. Databases such as MOD-finder, and CEBS facilitate finding published omics datasets and TOXsIgN, and the Comparative Toxicogenomics Database (CTD) facilitate submission, storage, and retrieval of cross-species toxicogenomic signatures based on molecular function [92,93,94,95,96]. Ideally, however, a curated database containing relevant information such as raw sequences of differentially expressed genes, proteins, etc. would further accelerate the quest for identifying functional bridging effects. To date, 412,969 organisms have been completely or partially sequenced and are available via GOLD (Genome OnLine Database; http://www.genomesonline.org, accessed on 1 August 2021). Initiatives such as i5K, the Darwin Tree of Life Project (https://www.darwintreeoflife.org), the Vertebrate Genomes Project (https://vertebrategenomesproject.org), the Fish10K project, and the Earth BioGenome Project are making the application of omics increasingly accessible for a multitude of hitherto unstudied species [97,98,99]. A narrow focus on only a handful of species may constrain our understanding of chemical-induced disease processes and molecular systems that have evolved to respond to stressor exposures and environmental change [100]. The continued study of non-model species and the integration between phylogenetically diverse datasets may pave the way for determining the effects an anthropogenically impacted and rapidly changing environment has on ecologically-relevant species and the populations they form in nature [101,102,103,104].

## 5. Conclusions

The sheer amount of publicly available data on tens of thousands of genes and their products calls for integrative analyses of cross-species responses. A focus on identifying the members and functions of evolutionarily conserved pathways and expression signatures that repeatedly emerge from differential and network modeling analyses across different stressors and species may lead to a redefinition of how omics technologies are being applied in ecotoxicology and stress ecology. Streamlining the use of omics technologies in risk assessment and environmental monitoring requires an expanded reference database and a better understanding of the relationships between specific responses and the functions of identified biomarker patterns. Comparing and systematizing repeatedly identified pathways will be a crucial step if we are to understand and predict the impact of existing and novel stressors and their mixtures on underrepresented ecosystems and species. Ecotoxicologists and stress ecologists are still faced with the daunting task of potentially evaluating thousands of stressors and species. Although the application of omics in ecotoxicology has many challenges [105], there is increased integration of different omics levels, a growing availability of genomic information on non-model species, an increasing number of public databases curating information across studies, an improved arsenal of bioinformatics, and computational tools, and an increasing variety of stressors and non-model species investigated.

## Data Availability

The dataset supporting the conclusions of this article are included in Supporting Information File 2 and can be used to build upon the findings.

## Supplementary Materials

The following data are available online at https://www.mdpi.com/article/10.3390/genes12101481/s1, Figure S1: Supporting Information File 1. Table S1: Dataset used in this review.
