# Supplementary material for: Trends in the Application of “Omics” to Ecotoxicology and Stress Ecology
Source: Genes (Basel). 2021 Sep 23;12(10):1481. doi: 10.3390/genes12101481 (PMC8535992; doi:10.3390/genes12101481)
Supplement: Supplementary file 1 [file genes-12-01481-s001.zip › SI1.pdf]

Supporting Information file 1 (SI1) for

Trends in the Application of "Omics" to Ecotoxicology and Stress Ecology

Joshua Niklas Ebner [1]

[1] Spring Ecology Research Group, Department of Environmental Sciences, University of Basel, 4056 Basel, Switzerland; joshua.ebner@unibas.ch

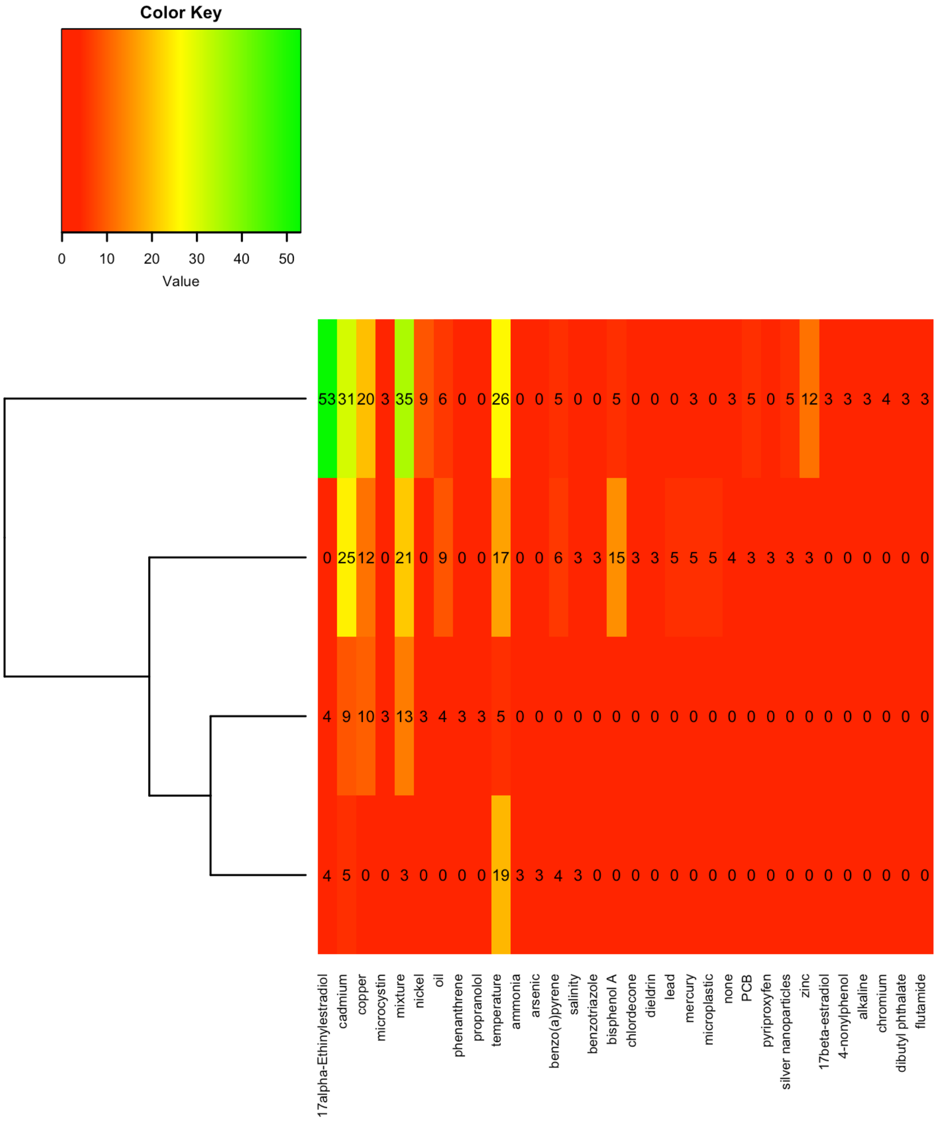

SI1 Figure S1: Heatmap showing the number of times the adverse effects of a specific stressor has been investigated using one of the four omics methods.
